# *Mycobacterium tuberculosis* Rv3628 drives Th1-type T cell immunity via TLR2-mediated activation of dendritic cells and displays vaccine potential against the hyper-virulent Beijing K strain

**DOI:** 10.18632/oncotarget.8771

**Published:** 2016-04-16

**Authors:** Woo Sik Kim, Jong-Seok Kim, Seung Bin Cha, Hongmin Kim, Kee Woong Kwon, So Jeong Kim, Seung Jung Han, Soo Young Choi, Sang-Nae Cho, Jong-Hwan Park, Sung Jae Shin

**Affiliations:** ^1^ Department of Microbiology, Institute for Immunology and Immunological Diseases, Brain Korea 21 PLUS Project for Medical Science, Yonsei University College of Medicine, Seoul, South Korea; ^2^ Laboratory of Animal Medicine, College of Veterinary Medicine, Chonnam National University, Gwangju, South Korea

**Keywords:** tuberculosis, DC maturation, Toll-like receptor 2, multifunctional T cell, subunit vaccine, Immunology and Microbiology Section, Immune response, Immunity

## Abstract

Identification of vaccine target antigens (Ags) that induce Ag-specific Th1 immunity is the first step toward the development of a tuberculosis vaccine. Here, we evaluated the *Mycobacterium tuberculosis* (Mtb) protein Rv3628, a soluble inorganic pyrophosphatase, as a vaccine target and characterized the molecular details of its interaction with dendritic cells (DCs). Rv3628 activated DCs, increasing their expression of cell surface molecules and augmenting their production of TNF-α, IL-1β, IL-6, and IL-12p70. Rv3628 mediated these effects by binding to TLR2 and activating downstream MyD88-, MAPK- and NF-κB-dependent signaling pathways. Rv3628-stimulated DCs induced the expansion of OVA-specific CD4^+^ and CD8^+^ T cells, which secreted IFN-γ and IL-2. Rv3628-specific effector/memory T cells expanded to a similar extent as those stimulated with ESAT-6 Ag in samples of lung and spleen cells collected from Mtb-infected mice. Finally, an Rv3628 subunit vaccine adjuvanted with dimethyldioctadecylammonium liposomes containing monophosphoryl lipid-A caused significant reductions in bacterial counts and lung inflammation after challenge with the hyper-virulent Mtb K strain. Importantly, protective efficacy was correlated with the generation of Rv3628-specific CD4^+^ T cells co-producing IFN-γ, TNF-α and IL-2 and exhibiting an elevated IFN-γ recall response. Thus, Rv3628 polarizes DCs toward a Th1 phenotype and promotes protective immunity against Mtb infection.

## INTRODUCTION

Tuberculosis (TB) caused by *Mycobacterium tuberculosis* (Mtb) remains a prevalent health threat worldwide [1-3]. The *Mycobacterium bovis* bacillus Calmette-Guérin (BCG) vaccine, the only currently licensed vaccine against TB, has been in use for approximately a century and has helped to control the global TB burden; however, its protective efficacy gradually wanes over time, eventually leading to an inability to prevent pulmonary TB in adults [4]. Therefore, the development of more efficacious TB vaccines is a top priority in TB research.

The generation of a robust Th1-type CD4^+^ T cell response is pivotal in providing anti-TB immunity. Generally, T cells are primed and educated in draining lymph nodes by dendritic cells (DCs) and consequently migrate to infected tissues to combat Mtb. Thus, DCs play key roles in programming and establishing T cell memory responses by translating innate immunity into immunological memory [5]. In the context of vaccine development, the initial encounter between DCs and an antigen (Ag) is the first critical event that shapes the type and duration of an immune response [1, 2]. Thus, an Ag that can induce DC maturation and consequently induce robust cellular immunity is of great interest for the development of an effective TB vaccine.

Previously, our group sought to identify suitable vaccine Ag targets with the aim of developing a multistage vaccine [6-9]. We have characterized many well-known and lesser-known Ags *in vitro*, particularly focusing on their interactions with DCs. While screening Ag-DC interactions, we discovered that Rv3628 induced a Th1-biased immune response via DC activation. Upon evaluating the Ag-DC interaction, we identified Rv3628 as a strong DC activator.

Rv3628 is a unique soluble inorganic class I pyrophosphatase (PPase) that, as a metal-dependent enzyme that converts pyrophosphate into orthophosphate, is essential for maintaining cell viability [10, 11]. In addition, in contrast to the PPases of other bacteria, Rv3628 contains two non-conserved amino acid residues, His21 and His86, in its active site [11]. This protein has been suggested as a possible target for the rational design of anti-TB agents [12]. Rv3628 has been identified in culture supernatants, membranes, cytosolic fractions and cell wall fractions of Mtb [13-16]. Rv3628 is constitutively expressed and is important for the survival and growth of Mtb *in vitro*; in particular, it is an essential factor that is up-regulated in dormant phages [17-19]. Although Rv3628 expression is not stress-related, one previous study showed that Rv3628 exhibited 4-fold greater expression in activated macrophages compared to resting macrophages [20]. A better understanding of the mycobacterial protein Rv3628 and its role in the host immune response may facilitate the rational design of more effective vaccines, such as multi-antigenic Mtb subunit vaccines.

Vaccine efficacy clearly depends on Mtb strain type [21]. In particular, the Mtb Beijing lineage is one of the most transmissible clades worldwide and exhibits epidemiological dominance in East Asia [22]. The Mtb K strain, which phylogenetically belongs to the Beijing lineage, was first isolated in South Korea in 1998, when a severe outbreak of pulmonary TB occurred in senior high schools [22]. This strain showed a highly virulent phenotype in a murine model of TB. This phenotype included rapid multiplication during the early stage of infection and excessive lung inflammation, and infection with this strain resulted in more rapid death of mice than infection with a reference H37Rv strain [22].

Overall, effective Ag targets for the rational design of Mtb vaccines should satisfy the following requirements: constitutive expression, accessibility (cell wall-associated or secreted) to Ag-presenting cells (APCs), ability to activate DCs, ability to be recognized by the immune system during *in vivo* infection, ability to induce a Th1-biased memory immune response, and efficacy against hyper-virulent Mtb strains. In this study, we evaluated Rv3628, a vaccine candidate that fulfills these criteria and is effective against challenge with the highly virulent Mtb K strain. Additionally, we investigated the molecular details underlying the interactions formed between this Ag and DCs.

## RESULTS

### Purification and cytotoxicity assay of recombinant Rv3628

We first purified Rv3628 under endotoxin-free experimental conditions. To remove any contaminating endotoxins, the purified Rv3628 was exposed to polymyxin B agarose. The expected molecular weight of Rv3628 is approximately 19 kDa, and its size was confirmed by SDS-PAGE and Western blotting (Supplementary Figure S1A). Next, we examined whether Rv3628 is cytotoxic to DCs (Supplementary Figure S1B). Rv3628 was not cytotoxic to DCs at a concentration of 10 μg/ml, indicating that a concentration below 10 μg/ml would not interfere with the subsequent experiments.

### Rv3628 protein induces functional and phenotypic maturation of DCs

**Figure 1:**
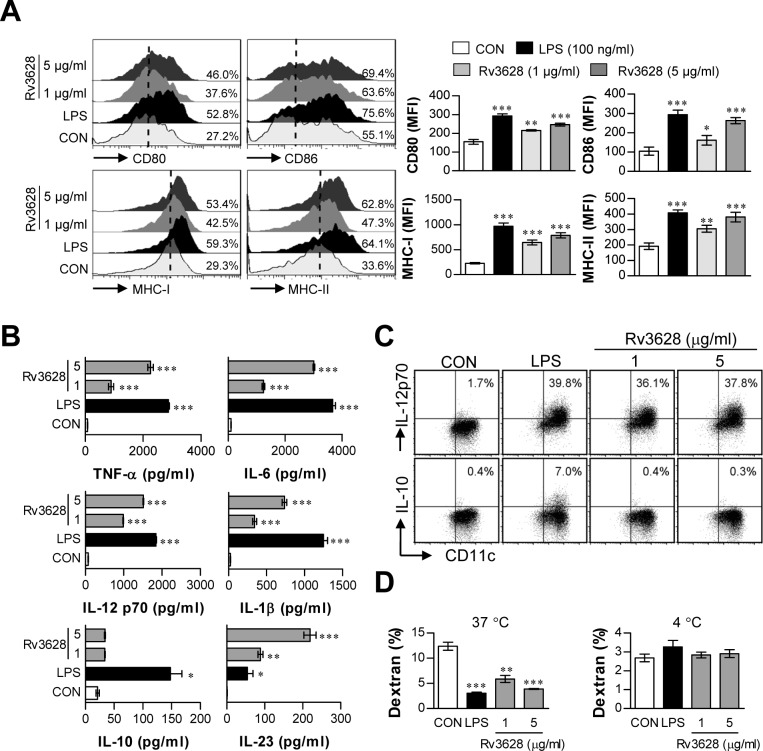
Rv3628 induces DC maturation in a dose-dependent manner Eight-day-old, immature DCs were treated with the indicated concentrations of Rv3628 or LPS for 24 h. **A.** DCs were stained with anti-CD80, anti-CD86, anti-MHC class I, or anti-MHC class II mAbs and analyzed for the expression of surface markers. The median fluorescence intensity (MFI) of the positive cells is shown for each panel. **B.** TNF-α, IL-6, IL-1β, IL-10, IL-23 and IL-12p70 levels in the culture medium were measured by ELISA. **C.** Dot plots of intracellular IL-12p70 and IL-10 in CD11c^+^ DCs. **D.** Endocytic activity was assessed at 37°C or 4°C by flow cytometric analysis of dextran-FITC uptake. The percentages of dextran-FITC-positive and CD11c^+^-positive cells are indicated. All bar graphs show the means ± SD of 3 samples. One representative plot out of three independent experiments is shown; *p < 0.05, **p < 0.01, and ***p < 0.001 for the treatments compared with untreated DCs (CON).

To investigate whether Rv3628 protein induces DC activation, we first measured the expression of phenotypic markers of DC maturation by flow cytometry. To accomplish this, DCs were treated with either lipopolysaccharide (LPS, 100 ng/ml) as a positive control or Rv3628 (1 or 5 μg/ml) for 24 h. We found that Rv3628 significantly augmented the expression of CD80, CD86, MHC class I molecules, and MHC class II molecules in a dose-dependent manner (Figure 1A). To examine the functional activation of DCs by Rv3628, we next examined the secretion of pro- and anti-inflammatory cytokines. Rv3628 significantly increased DC secretion of TNF-α, IL-6, IL-1β and IL-23 in a dose-dependent manner (Figure 1B). We then investigated the production of IL-12p70 and IL-10, which stimulate the proliferation and development of Th1 and Th2 cells, respectively. Interestingly, Rv3628 significantly induced the production of IL-12p70, but not that of IL-10 (Figure 1B and 1C). Because the capacity of DCs to take up an Ag (e.g., dextran) decreases during DC maturation after Ag recognition, we next investigated the role of Rv3628 in DC endocytosis. As shown in Figure 1D, the endocytic activity of Rv3628-treated DCs was significantly decreased to a similar extent to that of LPS-treated DCs. These experiments were repeated at 4°C, and the results showed that the uptake of dextran-FITC by DCs was inhibited at a low temperature. Thus, the reduced endocytic activity of the Rv3628-treated DCs was indicative of their increased functional maturity. These results strongly indicate that Rv3628 phenotypically and functionally activates DCs and polarizes these cells toward a Th1 response.

### Confirmation of LPS decontamination in purified Rv3628 samples

Because Rv3628-induced DC maturation may have resulted from contamination with LPS derived from *E. coli* during protein preparation, we confirmed the lack of endotoxin contamination in the *E. coli*-derived Rv3628 protein isolates using an Endpoint Chromogenic Limulus Amebocyte Lysate Assay kit (Lonza, Walkersville, MD, USA). We also assessed the effects of proteinase K pretreatment and heat denaturation. The levels of endotoxin in the Rv3628 preparations were < 15 pg/ml (< 0.1 EU/ml, which is the lowest detectable level) (data not shown). Furthermore, proteinase K pretreatment and heat denaturation abrogated the ability of Rv3628 to trigger DC maturation (Supplementary Figure S2A and S2B). These results revealed that DC maturation was directly induced by intact Rv3628 and was not caused by potential endotoxin contamination.

### Rv3628 induces DC activation by binding to TLR2 and activating the downstream MyD88 signaling pathway

**Figure 2:**
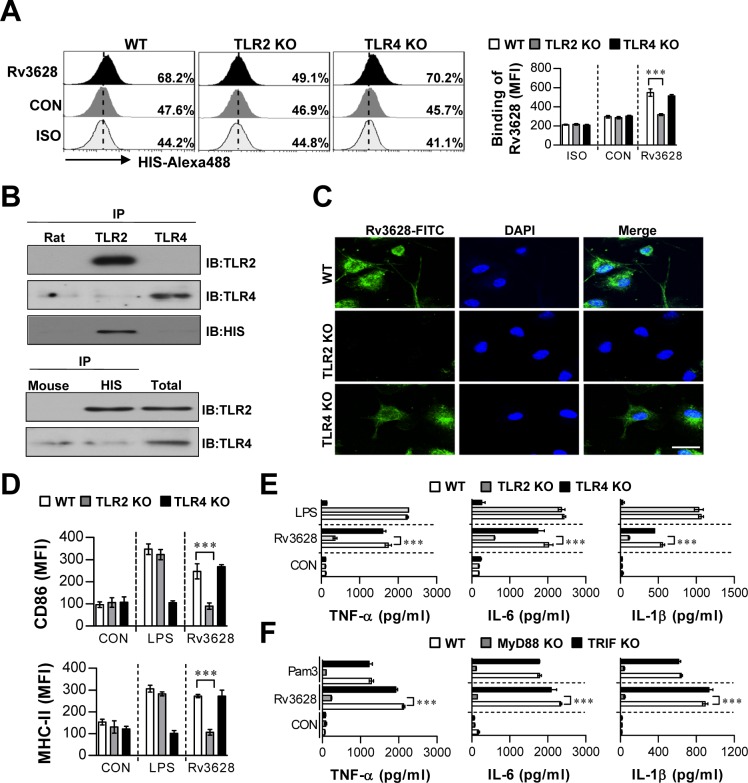
Rv3628 induces DC activation by interacting with TLR2 **A.** Bone marrow-derived DCs (BMDCs) from WT, TLR2 KO, and TLR4 KO mice were treated with Rv3628 for 1 h and stained with an Alexa488-conjugated anti-His mAb. The MFI of the positive cells is shown in each panel. The bar graphs show the means ± SEM of the percentages of Rv3628-treated Alexa488-positive cells among the CD11c^+^ cells from three independent experiments. ***p < 0.001 compared with Rv3628-treated WT DCs. **B.** DCs were treated with Rv3628 for 6 h. The cells were harvested, and cell lysates were immunoprecipitated with anti-rat IgG, anti-mouse IgG, anti-His, anti-TLR2, or anti-TLR4. Proteins were visualized by immunoblotting with anti-His, anti-TLR2, or anti-TLR4 Abs. One representative plot out of three independent experiments is shown. **C.** Fluorescence intensities reflecting Rv3628 binding to Rv3628-treated DCs. DCs derived from WT, TLR2 KO, and TLR4 KO mice were treated with Rv3628 for 1 h, fixed, and stained with DAPI and a FITC-conjugated anti-Rv3628 Ab. One representative plot out of three independent experiments is shown. **D.** and **E.** DCs derived from WT, TLR2 KO, and TLR4 KO mice were treated with Rv3628 and LPS (100 ng/ml) for 24 h. The bar graphs show the regulation of surface molecules and pro-inflammatory cytokines among CD11c^+^-gated Rv3628-treated DCs derived from WT, TLR2 KO, and TLR4 KO mice. **F.** DCs derived from WT, MyD88 KO, and TRIF KO mice were treated with Rv3628 and a MyD88-dependent TLR2 agonist (Pam3, 100 ng/ml) for 24 h. TNF-α, IL-6, and IL-1β production in Rv3628- or Pam3-treated DCs derived from WT, MyD88 KO, and TRIF KO mice was measured by ELISA. All data are expressed as the means ± SD of 3 samples. One representative plot out of three independent experiments is shown. **p < 0.01 and ***p < 0.001 compared with Rv3628-treated WT DCs. CON: untreated DCs; ISO: isotype control.

It is well known that the host immune response is initiated in response to interactions between pattern recognition receptors, such as TLRs, and pathogen-associated molecular patterns (PAMPs) on secreted Mtb Ags and cell wall components [23]. Thus, we examined whether Rv3628 can be recognized by and act through TLRs in DCs. To identify the TLRs on DCs that interact with Rv3628, WT-, TLR2 KO-, and TLR4 KO-derived DCs were stimulated with Rv3628, and Rv3628 on the cell surface was detected with an Alexa488-conjugated anti-His mAb. Rv3628 bound to the cell surface of WT- and TLR4 KO-derived DCs, but not that of TLR2 KO-derived DCs (Figure 2A). To confirm this interaction between Rv3628 and TLRs, we performed immunoprecipitation studies with TLR2 or TLR4 and Rv3628 in DCs. We found that Rv3628 bound to TLR2, but not to TLR4 (Figure 2B). This result was confirmed by confocal microscopy, which showed that Rv3628 bound to TLR2 molecules on the cell surface, but not to TLR4 molecules, as expected (Figure 2C). To further confirm the role of TLR2 in Rv3628-mediated DC maturation, we determined the expression of surface molecules and the production of cytokines in DCs isolated from WT, TLR2 KO and TLR4 KO mice. We found that Rv3628 induced the expression of various surface molecules (Figure 2D) and augmented the secretion of pro-inflammatory cytokines (Figure 2E) in WT- and TLR4 KO-derived DCs compared to TLR2 KO-derived DCs.

Within the TLR family, TLR2, TLR3, TLR4, TLR7 and TLR9 and their adaptor molecules MyD88 and TRIF play the most prominent roles in provoking innate immune responses [24]. Thus, MyD88 and TRIF have the potential to control various TLR-mediated signaling pathways. To investigate the relevance of the MyD88- and TRIF-dependent pathways to Rv3628-induced cytokine production by DCs, we compared cytokine secretion in WT-, MyD88 KO- and TRIF KO-derived DCs. As expected, the Rv3628-induced production of TNF-α, IL-6, and IL-1β was significantly reduced in MyD88 KO mice, but not in TRIF KO mice (Figure 2F). Collectively, these data suggest that Rv3628-induced DC maturation is predominantly mediated via TLR2/MyD88-mediated signaling pathways.

### Activation of the NF-κB and MAPK pathways mediates Rv3628-induced DC maturation

**Figure 3:**
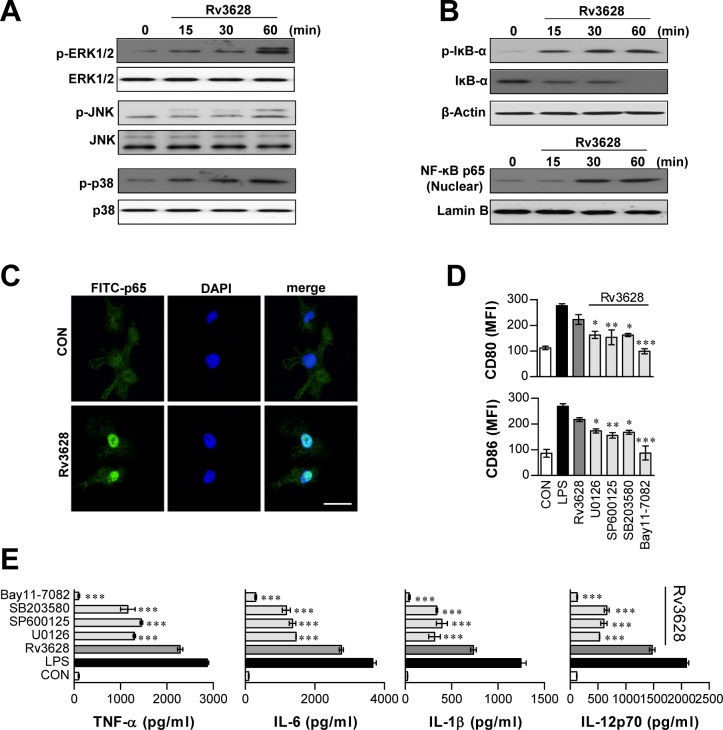
Rv3628-dependent induction of DC maturation protein involves activation of the MAPK and NF-κB signaling pathways **A.** and **B.** DCs treated with Rv3628 (5 μg/ml) for the indicated periods. Cell lysates were subjected to SDS-PAGE, and immunoblot analysis was performed using specific Abs against phospho-p38 (p-p38), p38, phospho-ERK1/2 (p-ERK1/2), ERK1/2, phospho-JNK (p-JNK), JNK, phospho-IκB-α (p-IκB-α), IκB-α, and p65 NF-κB. The results of one representative experiment out of three experiments producing similar results are shown. **C.** The effect of Rv3628 on the cellular localization of the p65 subunit of NF-κB in DCs. DCs were plated on glass chamber slides and treated with Rv3628 for 1 h. After Ag stimulation, the immunoreactivity of the p65 subunit of NF-κB in the DCs was analyzed by immunofluorescence, as described in the *Materials and Methods* section (original scale bar: 5 μm). The results of one representative experiment out of three experiments producing similar results are shown. **D.** and **E.** DCs were pretreated with different pharmacological inhibitors, such as SB203580 (p38 inhibitor), U0126 (ERK inhibitor), SP600125 (JNK inhibitor), or Bay11-7082 (NF-κB inhibitor) for 1 h prior to treatment with Rv3628 for 24 h; DMSO served as a vehicle control. D. CD80 and CD86 expression was analyzed by flow cytometry. E. TNF-α, IL-6, IL-1β and IL-12p70 levels in the culture medium were measured by ELISA. The data points shown are the means ± SD of 3 samples. One representative plot out of three independent experiments is shown; *p < 0.05, **p < 0.01 and ***p < 0.001 compared to treatment with Rv3628 alone. CON: untreated DCs.

Next, we investigated the downstream pathways of TLR2-MyD88, specifically, the MAPK and NF-κB pathways, because these pathways play critical roles in mediating cellular responses [25]. We specifically hypothesized that the Rv3628-induced maturation of DCs involves the NF-κB and MAPK signaling pathways. As shown in Figure 3A, Rv3628 triggered the phosphorylation of MAPKs such as ERK, JNK, and p38 MAPK in DCs. Moreover, we found that Rv3628 induced the phosphorylation and degradation of inhibitor of κB (IκB)-α and the nuclear translocation of p65 from the cytosol (Figure 3B and 3C). To understand the functional roles of these kinases in the Rv3628-induced activation of DCs, we used highly selective kinase inhibitors and examined their effects on Rv3628-induced DC maturation. In particular, DCs were pretreated for 1 h with or without inhibitors of p38, ERK1/2, JNK, or NF-κB. As expected, we found that these pharmacological inhibitors significantly abrogated the Rv3628-induced expression of the co-stimulatory molecules CD80 and CD86 on the surfaces of DCs (Figure 3D) and augmented the production of pro-inflammatory cytokines such as TNF-α, IL-6, IL-1β and IL-12p70 (Figure 3E). These data suggest that the NF-κB and MAPK signaling pathways are critically important for optimal Rv3628-induced DC activation, triggering the secretion of pro-inflammatory cytokines and the expression of phenotypic markers for DC maturation.

### Rv3628-stimulated DCs enhance naïve T cell proliferation and favor Th1 polarization

**Figure 4:**
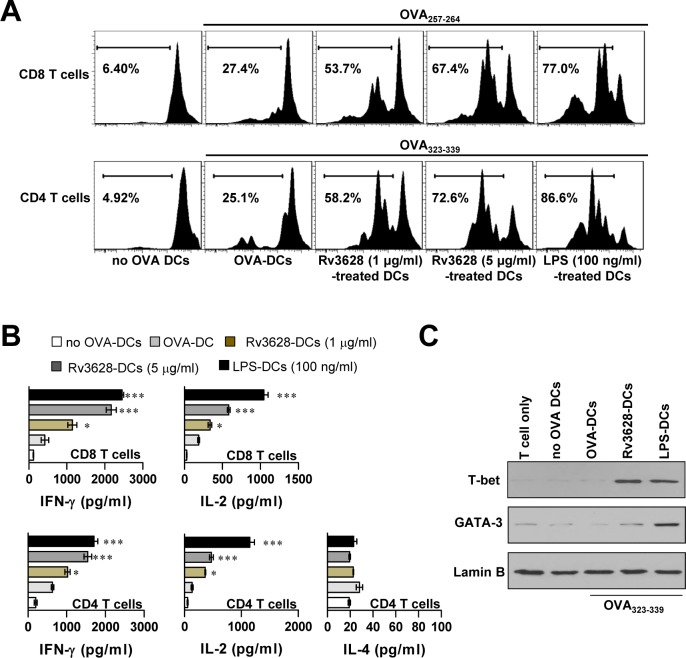
Rv3628-treated DCs stimulate T cells to produce Th1 cytokines T cell proliferation and T cell types were analyzed in OVA-specific mice as described in the *Materials and Methods* section. **A.** The proliferation of OVA-specific CD4^+^ and CD8^+^ T cells was assessed by flow cytometry. The results of one representative experiment out of three experiments producing similar results are shown. **B.** The culture supernatants obtained under the conditions described in part A were harvested after 24 h, and IFN-γ, IL-2 and IL-4 levels were analyzed by ELISA (top panel, OT-I; lower panel, OT-II). The data are shown as the means ± SD of 3 samples. One representative plot out of three independent experiments is shown. *p < 0.05, **p < 0.01, and ***p < 0.001 for comparisons with T cell/OVA_257-264_-pulsed DCs or T cell/OVA_323-339_-pulsed DCs. **C.** Western blot analysis of T-bet and GATA-3 expression in OVA-specific CD4^+^ T cells using specific anti-T-bet and anti-GATA-3 mAbs. The results of one representative experiment out of three experiments producing similar results are shown. no OVA-DCs: untreated DCs; OVA-DCs: OVA-treated DCs; Rv3628-DCs: Rv3628-treated DCs; LPS-DCs: LPS-treated DCs.

The primary roles of mature DCs are Ag presentation and subsequent interactions with T cells, which induce T cell polarization [26]. To precisely characterize the effects of Rv3628 on the interactions that form between DCs and T cells, we performed a syngeneic T cell proliferation assay using OT-I T cell receptor (TCR)-transgenic CD8^+^ T cells (OVA_257-264_) and OT-II TCR-transgenic CD4^+^ T cells (OVA_323-339_). To accomplish this, CFSE-labeled OVA-specific CD4^+^ and CD8^+^ T cells were co-cultured with Rv3628-treated DCs and pulsed with OVA_257-264_ or OVA_323-339_. These treated cells proliferated to a considerably greater extent than identical T cells co-cultured with non-Rv3628-treated DCs pulsed with OVA_257-264_ or OVA_323-339_ (Figure 4A). We then investigated the induction of cytokines (Figure 4B) and transcription factors (Figure 4C) in CD4^+^ and CD8^+^ T cells. IFN-γ and T-bet are expressed at high levels in Th1 cells, whereas IL-4 and GATA-3 are expressed at high levels in Th2 cells. As shown in Figure 4B and 4C, IFN-γ production and T-bet expression were both significantly higher in Rv3628-treated DCs than in untreated DCs; however, the levels of IL-4 and GATA-3 were not affected. These results suggest that Rv3628-treated DCs contribute to the polarization of naïve CD4^+^ and CD8^+^ T cells toward an IFN-γ-producing Th1-type T cell phenotype.

### Rv3628-TLR2 binding in DCs is essential for the generation of effector/memory T cells

**Figure 5:**
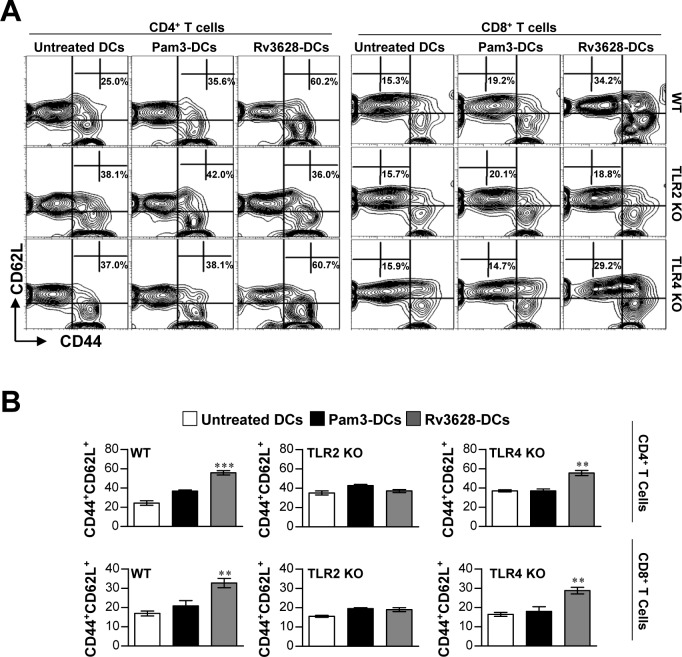
Rv3628 induced Ag-specific effector/memory T cell expansion in the spleens of Mtb H37Rv-infected mice via TLR2 signaling **A.** and **B.** WT-, TLR2 KO-, and TLR4 KO-DCs were treated for 24 h with Rv3628 (5 μg/ml) or Pam3 (100 ng/ml). Untreated DCs, Rv3628-treated DCs (Rv3628-DCs) and Pam3-treated DCs (Pam3-DCs) were co-cultured for 3 days with T cells of H37Rv-infected mice at a DC to T cell ratio of 1:10. The T cells were stained with anti-CD4, CD8, CD62L, and CD44 mAbs. **A.** Contour and **B.** bar graphs show CD62L^+^CD44^+^T cell populations in the harvested spleen cells. Bar graphs show the percentages of effector/memory T cells (CD4^+^CD44^+^CD62L^−^ and CD8^+^CD44^+^CD62L^−^) from one representative plot out of three independent experiments. The mean values ± SD of 4 samples are shown. Statistical significance (**p<0.01 or ***p<0.001) is indicated for the different treatments compared to untreated DCs.

Ag-matured DCs promote memory T cell expansion, differentiation and activation [26]. Therefore, we investigated whether DCs that underwent maturation following exposure to Rv3628 could specifically stimulate effector/memory CD4^+^ and CD8^+^ T cells isolated from the spleens of Mtb H37Rv-infected mice at 8 weeks post-infection. To accomplish this, we measured naïve T cell (CD44^low^CD62L^high^) and effector/memory T cell (CD44^high^CD62L^low^) subpopulations within CD4^+^ and CD8^+^ splenic T cell populations using flow cytometry. Splenic T cells collected from Mtb-infected mice were co-cultured with Rv3628-treated DCs derived from WT-, TLR2 KO-, or TLR4 KO-mice. As shown in Figure 5A and 5B, the Rv3628-treated WT and TLR4 KO DCs specifically induced the formation of effector/memory T cells, as evidenced by the significant quantities of CD44^high^CD62L^low^CD4^+^ T cells and CD44^high^CD62L^low^CD8^+^ T cells identified within the treatment group compared to the no-treatment group. In contrast, these subpopulations were not observed in T cell populations that were co-cultured with Rv3628-treated TLR2 KO-DCs. Taken together, these findings suggest that the interactions that form between Rv3628 and TLR2 are essential to the Rv3628-specific induction of effector/memory T cells.

### Recognition of Rv3628 by the immune system during the course of Mtb infection

**Figure 6:**
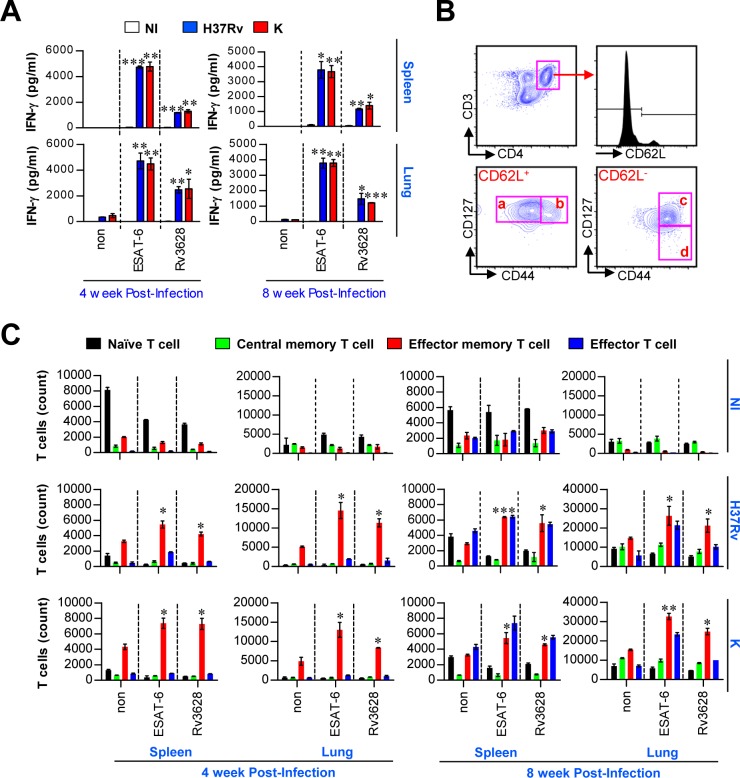
*Ex vivo*-stimulated Rv3628 induces Ag-specific IFN-γ production and memory T cell expansion in spleen and lung cells after challenge with Mtb strains **A**. Rv3628-specific IFN-γ production was analyzed in the spleen and lung cells of individual mice 4 and 8 weeks after challenge with aerosolized Mtb H37Rv or K. ESAT-6 was used as a positive control. The bar graphs show the means ± SD of 4 samples. One representative plot out of three independent experiments is shown. *p < 0.05, **p < 0.01, and ***p < 0.001. non: untreated cells; ESAT-6: ESAT-6-treated cells; Rv3628: Rv3628-treated cells. **B.** For the analysis of memory T cells, an inclusion gate was drawn around cells with equivalent forward scatter-height and forward scatter-area values to exclude doublets and larger cell aggregates (data not shown). CD4^+^ T cells were then gated based on their makers (CD3 and CD4), and these cells were further gated for naive cells (a: CD62L^+^CD127^+^CD44^−^), central memory cells (b: CD62L^+^CD127^+^CD44^+^), effector memory cells (c: CD62L^−^CD127^+^CD44^+^), and effector cells (d: CD62L^−^CD127^−^CD44^+^) within the CD3^+^CD4^+^ T cell population. **C.** At the same time point, spleen and lung cells were stimulated *in vitro* with Rv3628, and the numbers of naïve CD4^+^ T cells, effector CD4^+^ T cells, central memory CD4^+^ T cells and effector/memory CD4^+^ T cells were analyzed by flow cytometry. The data are expressed as the means ± SD of 4 samples from one representative experiment out of three independent experiments. Statistical significance (*p < 0.05, **p < 0.01, and ***p < 0.001) is shown for the treated cells compared to the non-treated effector/memory T cells. NI: non-infected mice, H37Rv: H37Rv-infected mice, K: K-infected mice.

The immunobiological potential of Rv3628 as a potent Mtb Ag that can be recognized by T cells was further investigated by examining Rv3628-induced IFN-γ production and memory T cell expansion in the spleens and lungs of Mtb H37Rv- and K-infected mice at various time points. Given that ESAT-6, which is produced by Mtb, is a representative T cell Ag in both mice and humans during infection [27], we used ESAT-6 as a positive control for the measurement of T cell activation and memory T cell generation in the Mtb-infected mice. As shown in Figure 6A, lung and spleen cells from both Mtb H37Rv- and K-infected mice showed significant IFN-γ production in response to Rv3628 or ESAT-6 stimulation. Next, the populations of memory T cells that were generated following stimulation with Rv3628 were analyzed by flow cytometry using a gating strategy (Figure 6B). Importantly, the lung and spleen cells that were stimulated with Rv3628 showed comparable levels of effector/memory T cell expansion, regardless of the Mtb strain used for infection (Figure 6C). These data suggest that Rv3628 is immunologically recognized during Mtb infection, causing a potent T cell response.

### Rv3628 immunization and analysis of immunogenicity

**Figure 7:**
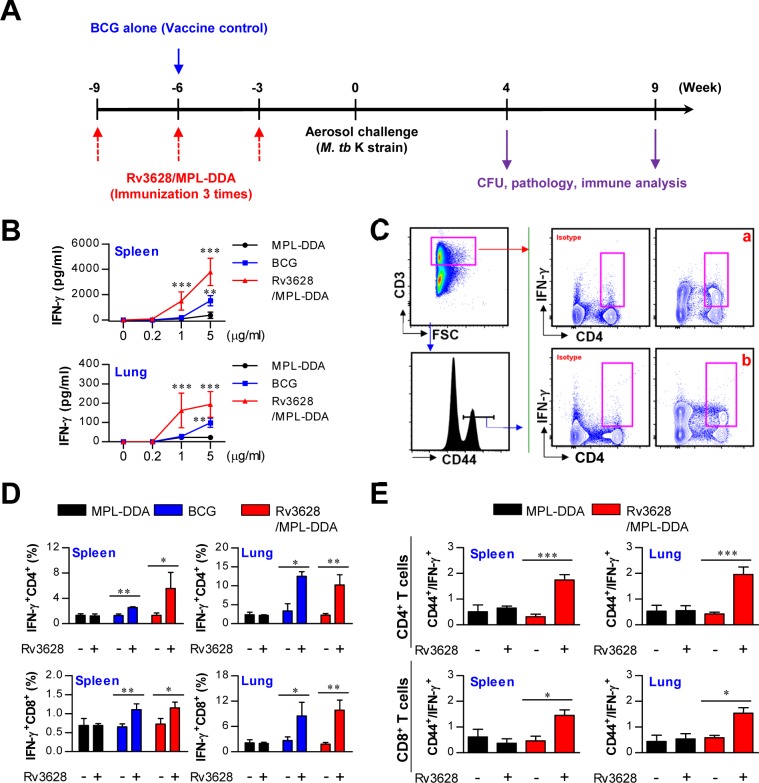
Ag-specific responses in spleen and lung cells after final immunization with MPL-DDA alone, BCG or Rv3628/MPL-DDA **A.** Schematic diagram of the experimental design. **B.** IFN-γ production by spleen and lung cells in response to Rv3628 stimulation was measured by ELISA. **p < 0.01 and ***p < 0.001 compared with the MPL-DDA-alone group **C.** to **E.** Staining panel and gating strategy used to identify IFN-γ-producing T cells (panel a) and Ag-specific IFN-γ-producing T cells (panel b) in spleen and lung cells from representative mice immunized with MPL-DDA, BCG or Rv3628/MPL-DDA. CD4^+^ and CD8^+^ T cells were identified based on CD3, CD4 and CD8 expression. Subsequently, IFN-γ-producing T cells (D: CD3^+^CD4^+^IFN-γ^+^ and CD3^+^CD8^+^IFN-γ^+^) and Ag-specific IFN-γ-producing T cells (E: CD3^+^CD4^+^CD44^+^IFN-γ^+^ and CD3^+^CD8^+^CD44^+^IFN-γ^+^) were gated as shown. *p < 0.05, **p < 0.01, and ***p < 0.001 compared with the Rv3628/MPL-DDA or BCG-immunized group. All graphs show the results from one of two experiments producing similar results (*n* = 6 animals/group).

The above results regarding Rv3628-mediated induction of T cell proliferation and Th1 response-mediated immunity motivated us to test the vaccine efficacy of Rv3628 following challenge with the highly virulent Mtb strain known as Beijing-K. Prior to this *in vivo* experiment, we investigated whether Rv3628 functions as an immunogenic protein when administered as an Ag in a subunit vaccine. For this purpose, we evaluated the induction of IFN-γ production, the generation of IFN-γ-producing T cells and Rv3628-specific IFN-γ-producing T cells, and the production of Ag-specific antibodies following immunization with a vaccine consisting of Rv3628 adjuvanted with MPL-DDA (Figure 7 and Supplementary Figure S3). To accomplish this, lung and spleen cells from an immunized mouse (immunized with MPL-DDA, BCG or Rv3628/MPL-DDA) were stimulated with Rv3628 at various doses (0.2, 1 or 5 μg/ml). We found that IFN-γ production significantly increased in the groups that were immunized with Rv3628/MPL-DDA upon cognate Rv3628 stimulation (1 μg/ml [*p* < 0.001] or 5 μg/ml [*p* < 0.001]) compared with the MPL-DDA-only groups (Figure 7B). Next, populations of IFN-γ-producing CD4^+^ T cells (panel a in Figure 7C) and Rv3628-specific IFN-γ-producing CD4^+^ T cells (panel b in Figure 7C) were restimulated with Rv3628 at a concentration of 5 μg/ml and then analyzed by flow cytometry using a gating strategy. IFN-γ-producing CD8^+^ T cells and Rv3628-specific IFN-γ-producing CD8^+^ T cells were also analyzed using the same gating strategy as in Figure 7C (data not shown). This restimulation significantly increased the subpopulations of IFN-γ-producing T cells (CD4^+^/IFN-γ^+^ and CD8^+^/IFN-γ^+^ cells, Figure 7D) and Ag-specific IFN-γ-producing T cells (CD4^+^/CD44^+^/IFN-γ^+^ and CD8^+^/CD44^+^/IFN-γ^+^ cells, Figure 7E) in the spleens and lungs of the Rv3628-immunized mice compared to the non-Rv3628-stimulated mice. Further analysis of Rv3628-specific antibody titers showed that the mice in the Rv3628-immunized groups exhibited significant Rv3628-specific IgG2c responses, but not IgG1 responses (Supplementary Figure S3). These results indicate that immunization with Rv3628 induces the development of effective Th1 immunity. Additionally, the BCG-immunized groups showed augmented IFN-γ release (Figure 7B) and increased populations of IFN-γ-producing T cells (Figure 7D) in response to stimulation with Rv3628 (5 μg/ml).

### Protective efficacy of the Rv3628 subunit vaccine against challenge with the Mtb K strain in a mouse model

**Figure 8:**
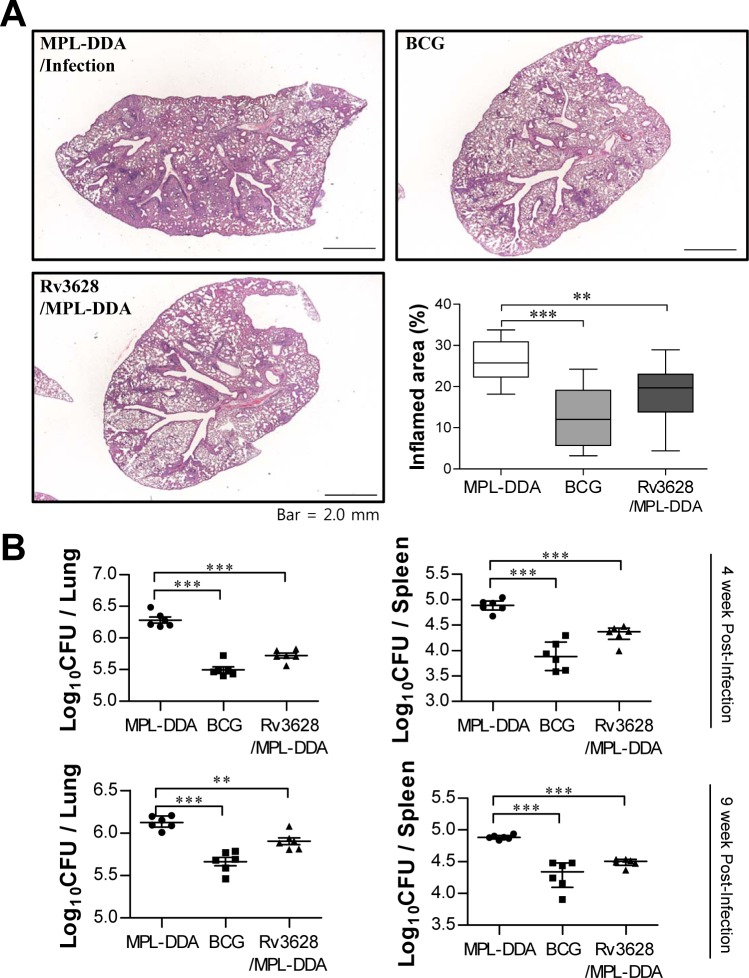
Histology of representative lung lobes and CFU values for each group **A.** Lung sections from each immunized mouse (immunization with MPL-DDA alone, BCG alone or Rv3628/MPL-DDA) were stained with H&E at 9 weeks after challenge with Mtb K. **B.** Differences in bacterial burden among mice immunized with BCG alone, Rv3628/MPL-DDA and those treated with the adjuvant control (MPL-DDA alone) at 4 and 9 weeks after challenge with Mtb K are shown. The results from one of two experiments producing similar results are shown (*n* = 6 animals/group). *p < 0.05, **p < 0.01, and ***p < 0.001 compared with the MPL-DDA-alone group.

We next assessed the level of protection that was generated against challenge with the Mtb K strain following immunization with Rv3628. To accomplish this, three weeks after the third immunization, the mice were challenged with an aerosol of the highly virulent Mtb K strain. At 4 and 9 weeks post-challenge, the MPL-DDA-, BCG- and Rv3628/MPL-DDA-immunized groups were histologically examined (Figure 8A and Supplementary Figure 4), and their bacterial burdens were quantified (Figure 8B). At 9 weeks post-challenge, the Rv3628/MPL-DDA-immunized group showed significantly reduced lung inflammation compared with the infection control group (*p* < 0.01); however, a lesser degree of pathological improvement following Rv3628 vaccination was observed at 4 weeks post-challenge compared to 9 weeks post-challenge (Supplementary Figure 4). In addition, compared with the infection control groups, the Rv3628-immunized mice exhibited significantly reduced bacterial burdens in the lungs and the spleen at 4 and 9 weeks post-challenge (Figure 8B).

### Maintenance of Rv3628-specific Th1 immune responses in mice infected with Mtb Beijing-K

**Figure 9:**
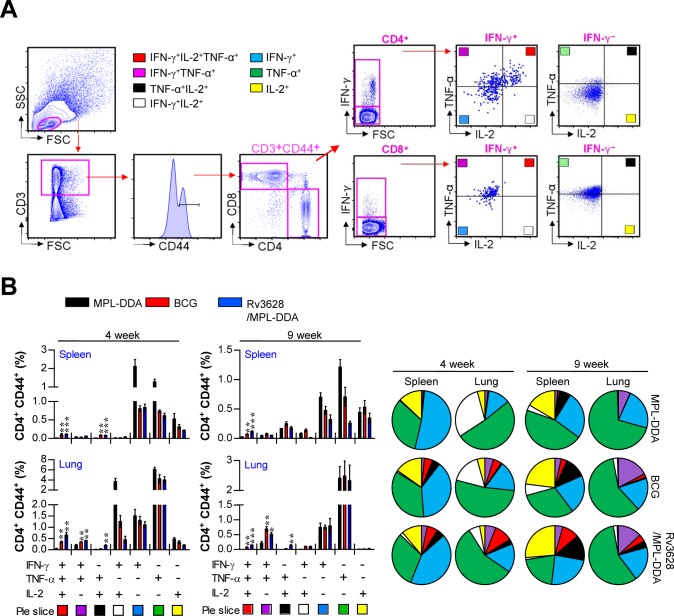
Rv3628/MPL-DDA induces the production of multifunctional CD4^+^ T cells **A.** and **B.** Cytokine production by Rv3628-specific CD4^+^ T cells in immunized mice (*n* = 6 animals/group) was analyzed at 4 and 9 weeks after challenge with Mtb K using flow cytometry. **A.** The strategy for gating multifunctional CD4^+^ T cells is shown for a representative mouse immunized with Rv3628/MPL-DDA. **B.** Spleen and lung cells from immunized mice were stimulated with Rv3628 (5 μg/ml) for 12 h in the presence of GolgiStop. Rv3628-stimulated cells were identified by intracellular cytokine staining based on CD3, CD4 and CD8 expression and were further gated for CD44^+^ cells. The percentages of cells expressing all three cytokines (IFN-γ, TNF-α, and IL-2), two of these three cytokines, or one of these three cytokines in each group are depicted in the bar graphs (B, left panel) and pie charts (B, right panel). The results of one representative study out of at least two independent studies are presented. *p < 0.05, **p < 0.01, and ***p < 0.001 compared with the MPL-DDA-alone group (unpaired t-test).

Th1-mediated immune responses and multifunctional T cells play important roles in generating protective immunity against Mtb [28, 29]. Therefore, we next evaluated how immunization with Rv3628 affected the T cell phenotype. At 4 and 9 weeks post-challenge, spleen and lung cells were stimulated *in vitro* with Rv3628, and the phenotypes of the responding CD4^+^ and CD8^+^ T cells were evaluated by multicolor intracellular cytokine staining and flow cytometry (Figure 9A). At 4 and 9 weeks post-challenge, increased levels of triple-positive CD4^+^ T cells (co-expressing IFN-γ, TNF-α and IL-2), but not CD8^+^ T cells, were observed in the spleens and lungs of the Rv3628-immunized group compared with the infection control group (Figure 9B and Supplementary Figure S5). In addition, the Rv3628-immunized group showed higher populations of double-positive multifunctional CD4^+^ T cells (IFN-γ^+^TNF-α^+^CD4^+^ and TNF-α^+^IL-2^+^CD4^+^ T cells) in the lung at 4 and 9 weeks post-challenge (Figure 9B), but this phenomenon was not observed for CD8^+^ T cells (Supplementary Figure S5).

### CD4^+^ T cell types induced by Rv3628 vaccination in mice infected with Mtb Beijing-K

Recent studies have shown that the development of Th1 and Th17 immune responses induces protection against TB [30]. In the present study, at 4 and 9 weeks post-challenge, T-bet-expressing CD4^+^ T cells (Th1-type cells), GATA-3-expressing CD4^+^ T cells (Th2-type cells) and RORγt-expressing CD4^+^ T cells (Th17-type cells) were evaluated by transcription factor staining and flow cytometry (Supplementary Figure S6A). T-bet-expressing CD4^+^ T cells were observed in the spleens and lungs of the Rv3628-immunized mice at 4 and 9 weeks post-challenge (Supplementary Figure S6B). Interestingly, immunization with Rv3628 induced the production of both T-bet-expressing CD4^+^ T cells and RORγt-expressing CD4^+^ T cells in the lung, but not in the spleen (Supplementary Figure S6B).

## DISCUSSION

In the current study, we first evaluated the functional roles assumed by Rv3628 when interacting with DCs. We found that Rv3628 acts as a selective agonist of TLR2 in DCs and functionally induces DC maturation by elevating the expression of cell surface molecules and the production of Th1-polarizing pro-inflammatory cytokines in DCs via the activities of the MyD88-, MAPK-, and NF-κB-dependent signaling pathways (Figures 1, 2 and 3). Furthermore, Rv3628 participates in adaptive immunity by shifting induced naïve T cell immune responses toward Th1 polarization (Figure 4). Although several previous reports have described the roles of TLR2 agonists produced by Mtb [30-32], to the best of our knowledge, this is the first time that the Mtb protein Rv3628 has been described as a TLR2 agonist.

In general, Mtb Ags that serve as TLR2 agonists—either as immune activators or as virulence factors—should be considered for inclusion in novel TB vaccines if they are recognized by the immune system [30-32]. For example, Mtb can expropriate the TLR2 signaling pathway to subvert host immunity by attenuating macrophage responses to IFN-γ, which suppresses Ag processing and presentation [33, 34]. Importantly, Rv1196, Rv1917c, and PPE18 have all been reported to interact with TLR2 molecules on macrophage and DC surfaces and to strongly induce the secretion of IL-10, which is known to favor a Th2 immune response [35, 36]. This immune response potentially serves as an immune evasion mechanism by blunting protective Th1 immunity against Mtb infection. As another example, the same group reported that LprG (Rv1411c) inhibits human macrophage-mediated MHC class II Ag processing, suppressing Ag recognition by CD4^+^ T cells [37]. However, Rv1196 and PPE18 are both components of the MF72F vaccine, which is currently under investigation in a Phase II clinical study. Furthermore, an Rv1411/ESAT-6 fusion protein has been shown to enhance vaccine efficacy in the absence of additional Th1 response-inducing adjuvants, indicating that this protein has natural adjuvanticity due to its interaction with TLR2 [32, 38, 39]. Alternatively, the Mtb Ag TB10.4, which is a TLR2 agonist, induces a strong Th1 response mediated by CD4^+^ T cells and displays good vaccine potential [40], and ESAT-6, the most fully characterized immunogenic Mtb Ag, promotes protective Th1 and Th17 responses in a TLR2-dependent manner [31]. These two Ags (TB10.4 and ESAT-6) are secreted during the infection course, and their common features are recognized by the host immune system [31, 40]. Thus, gaining an understanding of the cross-talk that forms between TLR2 and Mtb Ags will impact the design of novel therapeutic strategies and the development of vaccines and immunotherapy regimens.

Bertholet *et al*. specifically identified Rv3628 as a human T cell Ag [41]. When PBMCs from PPD-negative and PPD-positive healthy subjects were examined, Rv3628 was found to stimulate the PBMCs from all of the PPD-positive subjects, but not the PBMCs from any of the PPD-negative subjects. Additionally, an elevated IFN-γ recall response was observed in the Rv3628-stimulated PBMCs from the PPD-positive subjects [41]. In our study, IFN-γ production (Figure 6A) and CD4^+^ memory T cell expansion (Figure 6C) were observed in spleen and lung cells isolated from mice treated with Rv3628 at various time points after infection with Mtb strains (H37Rv and Beijing-K).

Interestingly, the protective efficacy induced by Rv3628 adjuvanted with CpG, a TLR9 agonist, was unexpectedly low in a murine TB model [41]. The MyD88 signaling pathway, which functions downstream of the adjuvant CpG, is shared by Rv3628. Importantly, a recent study showed that co-activation of the MyD88 and TRIF signaling pathways is an important mechanism supporting the development of sustained vaccine-induced protective T cell immunity against Mtb infection [42]. Thus, utilizing an Ag and an adjuvant that share the same signaling pathway may not be advantageous in TB subunit vaccine development, and an understanding of the nature of the Ag is important.

In the current study, we employed MPL as an adjuvant for the Rv3628 subunit vaccine. MPL appears to enable potent but safe adjuvanticity as a result of TRIF-biased signaling [43]. However, TRIF-biased TLR4 activation by MPL may not produce a maximal Th1 immune response. Indeed, synergistic interactions between MyD88 and TRIF are required for Th1 cell polarization by a TLR4-based adjuvant [44]. Accordingly, activation of both the MyD88 (by Rv3628) and TRIF (by MPL) signaling pathways may be essential for the protective efficacy of the Rv3628 subunit vaccine by enhancing Th1-biased immune responses against aerosol challenge with Mtb. In this context, the activation of both the MyD88 and TRIF signaling pathways is essential for effective adjuvant activity when an Ag displays self-adjuvanticity [44]. Thus, choosing an appropriate adjuvant or delivery platform is critical to the success of a subunit vaccine.

In general, high CD44 expression on T cell surfaces is considered to indicate that the T cells are antigen-experienced (i.e., they exhibit an antigen-specific effector/memory phenotype) [45]. In addition, a recent study showed that CD44 is essential to the generation and maintenance of memory T helper 1 (Th1) cells [46]. Interestingly, Rv3628/MPL-DDA-immunized mice showed increased populations of IFN-γ^+^CD44^+^ T cells (Figure 7E). Based on these results, we investigated the protective effect of the Rv3628/MPL-DDA vaccine against the Mtb K strain, a hyper-virulent W-Beijing-lineage strain, in a mouse model. Rv3628/MPL-DDA-immunized mice were substantially protected after challenge with the Mtb K strain based on assessments of lung pathology (Figure 8A and Supplementary Figure S4) and bacterial growth (Figure 8B).

Importantly, vaccine-induced protection against Mtb infection specifically involves the presence of Ag-specific multifunctional IFN-γ^+^TNF-α^+^IL-2^+^ and IFN-γ^+^TNF-α^+^ CD4^+^ T cells in the lungs [28, 47]. Additionally, Khader *et al*. showed that the induction of host-mediated Ag-specific IFN-γ and IL-17 responses induces significant protection against aerogenic challenge with both highly susceptible and highly resistant Mtb strains [48]. Importantly, an examination of Ag-immunized mice showed that immunization with Rv3628 significantly induced the production of multifunctional CD4^+^ T cells (Figure 9) in the lungs and the spleen following challenge with the Mtb K strain. Interestingly, CD4^+^ T cells from BCG-immunized mice showed weaker responses to Rv3628 than CD4^+^ T cells from Rv3628-immunized mice. However, the BCG-immunized mice showed substantial reductions in bacterial growth (slightly better than the Rv3628-immunized mice) despite the milder induction of Rv3628-specific T cell immunity compared to the Rv3628-immunized mice (Figure 8B and Figure 9B). These results suggest that BCG contains many Ags that can augment multifunctional T cell populations. In addition, the Rv3628-immunized mice exhibited early induction of T-bet^+^CD4^+^ T cells (Th1 cells) and RORγt^+^CD4^+^ T cells (Th17 cells) in the lungs following challenge with the Mtb K strain (Supplementary Figure S5). Based on these trends, Rv3628 can likely function as a central component of a successful vaccine against Mtb infection.

In summary, our data show that the Rv3628 protein plays a critical role in DC activation in a TLR2-dependent manner and induces the adaptive immune response by specifically promoting the development of naïve CD4^+^ T cells and the expansion of Ag-specific memory CD4^+^ T cells. Importantly, immunization with Rv3628 conferred protective immunity and imparted significant protection to mice infected with the Mtb K strain, indicating that Rv3628 is a potential candidate Ag for use in future vaccines against highly virulent Mtb strains.

## MATERIALS AND METHODS

### Ethics statement

All animal studies were performed in accordance with Korean Food and Drug Administration (KFDA) guidelines. The experimental protocols used in this study were reviewed and approved by the Ethics Committee and Institutional Animal Care and Use Committee (Permit Number: 2014-0197-3) of the Laboratory Animal Research Center at Yonsei University College of Medicine (Seoul, Korea).

### Animals

Wild-type (WT), Toll-like receptor (TLR) 2 knockout (KO), TLR4 KO, OT-I and OT-II T cell receptor (TCR) transgenic mice (C57BL/6 background) were purchased from Jackson Laboratory (Bar Harbor, ME, USA) and were maintained under barrier conditions in a BL-3 biohazard animal facility at the Yonsei University Medical Research Center in an environment with a constant temperature (24±1°C) and humidity (50±5%). The animals were fed a sterile commercial mouse diet and were provided with water ad libitum under standardized light-controlled conditions (12-h light and dark periods). The mice were monitored daily, and none of the mice exhibited any clinical symptoms or illness during the experimental period.

### Purification of recombinant Rv3628 protein from *Escherichia coli*

To produce recombinant Rv3628 protein, the corresponding gene was amplified by PCR using Mtb H37Rv ATCC 27294 genomic DNA as a template and the primer sequences 5′-CAATTCGACGTGACCATCGAA-3′ and 3′-GTGTGTACCGGCCTTGAAGCG-5′. The *rv3628* gene was inserted into a pET22b (+) plasmid (Novagen, Madison, WI), and the resultant products were sequenced. Plasmids containing recombinant Rv3628 were transformed into *E. coli* BL21 cells by heat-shock for 1 min at 42°C. After cell disruption via sonication, recombinant Rv3628 was purified using Ni-NTA resin as previously described with slight modifications [8]. To remove endotoxins, the dialyzed recombinant protein was incubated with polymyxin B-agarose (Sigma, St. Louis, MO, USA) for 6 h at 4°C. Finally, the purified endotoxin-free recombinant protein was filter-sterilized and frozen at -70°C. The protein concentration was estimated using a bicinchoninic acid protein assay kit (Pierce, Rockford, IL, USA) with bovine serum albumin as a standard.

### Generation and culture of DCs

Bone marrow-derived DCs (BMDCs) were prepared and cultured as previously described [8]. On day 8, over 80% of nonadherent cells expressed CD11c. To obtain highly purified cell populations for subsequent analyses, the DCs were labeled with a bead-conjugated anti-CD11c mAb (Miltenyi Biotec, Bergisch Gladbach, Germany), followed by positive selection on paramagnetic columns (LS columns; Miltenyi Biotec, Bergisch Gladbach, Germany) according to the manufacturer's instructions. The purity of the selected cell fraction was > 90%.

### Cytotoxicity analysis and Ag uptake assay

To investigate the cytotoxic effect of Rv3628 on the DCs, Rv3628 (5 μg/ml) was added to cultures of isolated DCs (1 × 10^6^ cells/ml), and DC cell death was analyzed. After 24 h of treatment, the DCs were harvested, washed with PBS and stained with FITC-Annexin V and propidium iodide (BD PharMingen, San Jose, CA, USA). Thereafter, DC cytotoxicity was analyzed using a FACSverse flow cytometer (Becton Dickinson, San Jose, CA, USA). The Ag-uptake ability of the DCs was determined according to a procedure reported by Kim *et al*. [8].

### Flow cytometry analysis of cell surface molecule expression

On day 8, the BMDCs were harvested, washed with PBS, and resuspended in fluorescence-activated cell sorter washing buffer (2% FBS and 0.1% sodium azide in PBS). The cells were stained with PE-labeled anti-H-2Kb (MHC class I) and anti-I-Ab (MHC class II), APC-labeled anti-CD80 and anti-CD86, and PE-Cy7-labeled anti-CD11c from eBioscience (San Diego, CA, USA) for 45 min at 4°C. The cells were washed three times with PBS and resuspended in PBS. The resulting fluorescence was measured via flow cytometry using FlowJo software (BD Biosciences). LPS (100 ng/ml) was used as a positive control for DC maturation.

### Cytokine assays

A sandwich enzyme-linked immunosorbent assay (ELISA) was used to detect IL-6, IL-1β, TNF-α, IL-12p70, IL-10, IL-17A, IL-4, IL-23 (eBioscience) and IFN-γ (BD Biosciences) levels in culture supernatants as previously described [7].

### Flow cytometric analysis and intracellular cytokine staining

To create single-cell suspensions, harvested spleens and lungs were incubated in RPMI 1640 digestion media (10% fetal bovine serum, 0.1% collagenase type II (Worthington), 1 mM MgCl_2_, and 1 mM CaCl_2_) at 37°C for 30 min. The single-cell suspensions were then filtered through a 40-μm cell nylon mesh cell strainer, treated with RBC lysis buffer (Sigma) for 5 min, and washed twice with RPMI 1640 medium supplemented with 2% FBS. Single-cell suspensions from the spleens and the lungs of immunized mice were stimulated with Rv3628 (5 μg/ml) for 12 h at 37°C in the presence of GolgiStop (eBioscience). The cells were first blocked with Fc Block (anti-CD16/32; eBioscience) for 15 min at 4°C and then stained with fluorochrome-conjugated antibodies against CD4, CD8, CD62L, CD44, CD127 (eBioscience) and CD3 (BD bioscience) for 30 min at 4°C. Cells stained with appropriate isotype-matched immunoglobulins were used as negative controls. The cells were fixed and permeabilized using a Cytofix/Cytoperm kit (BD Biosciences) according to the manufacturer's instructions. Intracellular TNF-α, IL-2, T-bet, GATA-3, RORγt (eBioscience) and IFN-γ (BD bioscience) levels were detected with fluorescein-conjugated antibodies in a permeation buffer. The cells were analyzed with a FACSverse flow cytometer using FlowJo software.

### Immunoprecipitation

DCs (1 × 10^7^) were incubated in Rv3628 (10 μg/ml) for 6 h, and the cells were then pelleted and lysed with Pierce immunoprecipitation lysis buffer (Rockford, IL, USA). The cell lysates were then pre-cleared with protein A or G Sepharose beads for 2 h at 4°C overnight with rotation. Rv3628 and TLR2- and TLR4-associated proteins were immunoprecipitated by incubation in protein A or G Sepharose for 24 h at 4°C after incubation with control antibodies (Abs) for 1 h at 4°C: anti-rat IgG served as a control for anti-TLR2 and anti-TLR4 monoclonal Abs (mAbs), and anti-mouse IgG served as a control for the anti-His mAb (Santa Cruz Biotechnology). The beads were harvested, washed and boiled in 5X sample buffer for 5 min. Proteins were separated via 10% SDS-PAGE followed by transfer to polyvinylidene difluoride membranes (Millipore). The membranes were further probed with anti-TLR2, ant-TLR4, and anti-His Abs as indicated.

### Confocal laser scanning microscopy

WT-, TLR2 KO- and TLR4 KO-derived DCs were incubated overnight on poly-L-lysine-coated glass coverslips. After treatment with Rv3628, the cells were fixed in 4% paraformaldehyde, permeabilized in 0.1% Triton X-100, and blocked with 2% bovine serum albumin (BSA) in PBS containing 0.1% Tween-20 (PBS/T) for 2 h before incubation in 2% BSA in PBS/T containing an anti-His Ab for 2 h at room temperature. After washing with PBS/T, the cells were incubated with a FITC-conjugated secondary antibody in a dark room for 1 h and then stained with 1 μg/ml DAPI for 10 min at room temperature. Cell morphology and fluorescence intensity were observed using a confocal laser scanning microscope (Zeiss LSM510 Meta). Images were acquired using LSM510 Meta software and processed using an LSM imager.

### Immunoblotting analysis

After stimulation, cells were lysed in 100 ml of Pierce RIPA buffer supplemented with protease inhibitor cocktail. Immunoblotting was performed as previously described [8].

### Nuclear extract preparation

Nuclear extracts from cells were prepared as follows. Cells were treated with 100 ml of lysis buffer [10 mM HEPES (pH 7.9), 10 mM KCl, 0.1 mM EDTA, 0.5% Nonidet P-40, 1 mM dithiothreitol (DTT), and 0.5 mM PMSF] on ice for 10 min. After centrifugation at 4000 rpm for 5 min, the pellet was resuspended in 100 ml of extraction buffer [20 mM HEPES (pH 7.9), 400 mM NaCl, 1 mM EDTA, 1 mM DTT, and 1 mM PMSF] and incubated on ice for 30 min. After centrifugation at 12,000 rpm for 10 min, the supernatant containing the nuclear extracts was collected and stored at -80°C until use.

### Treatment of DCs with pharmacological inhibitors of signaling pathways

All pharmacological inhibitors used in this study were purchased from Calbiochem (San Diego, CA, USA). Dimethyl sulfoxide (Sigma) was added to the cultures at a 0.1% vol./vol. concentration as a solvent control. DCs were washed with PBS and pretreated with inhibitors in RPMI 1640 medium containing glutamine for 1 h prior to treatment with Rv3628 (5 μg/ml) for 24 h. The inhibitors were used at the following concentrations: U0126, 10 μM; SB203580, 20 μM; SP600125, 10 μM; and Bay11-7082, 20 μM. For all experiments involving inhibitors, the inhibitor concentration used was selected after careful titration experiments and assessments of DC viability using MTT assays.

### *In vitro* T cell proliferation assay

Responder T cells, which participate in allogeneic T cell reactions, were isolated from total mononuclear cells prepared from mouse spleens using a magnetic-activated cell sorting column. OVA-specific CD8^+^ and CD4^+^ T cells, which were classified as responder T cells, were obtained from the spleens of OT-I and OT-II mice, respectively. These T cells were stained with 1 μM CFSE (Invitrogen). DCs (2 × 10^5^ cells per well) treated with OVA peptide in the presence of 1 or 5 μg/ml Rv3628 for 24 h were co-cultured with CFSE-stained CD8^+^ and CD4^+^ T cells (2 × 10^6^) at a DC:T cell ratio of 1:10. On day 3 of co-culture, each T cell culture was stained with a Percp-Cy5.5-conjugated anti-CD4 or anti-CD8 Ab (eBioscience) and then analyzed using a flow cytometer. The supernatants were harvested, and cytokine production was analyzed by ELISA.

### Confirmation of lipopolysaccharide decontamination in Rv3628 samples

To characterize the physical and chemical nature of Rv3628, the isolate was heated for 15 min at 100°C or digested for 1 h at 37°C with soluble proteinase K (Sigma) at 5 μg/ml with Rv3628. After 24 h, the TNF-α and IL-6 levels in the BMDC supernatant were analyzed using ELISA.

### *In vivo* experiments, vaccination and challenge in mice

We performed animal infection studies to address two specific aims: 1) to investigate whether Rv3628 is immunologically recognized in the lungs and spleens of Mtb-infected mice during early and late infection, and 2) to investigate whether the Rv3628 subunit vaccine is effective against challenge with the hyper-virulent Beijing strain. To address these questions, we conducted animal experiments with Mtb H37Rv and/or Mtb Beijing-K at various time points post-infection. For aim #1, lung and spleen cells collected from Mtb H37Rv- or Mtb Beijing-K-infected mice at 4 and 8 weeks post-infection were stimulated with Rv3628 or 6-kDa early secretory antigenic target (ESAT-6; 5 μg/ml). *Ex vivo*-stimulated Rv3628-specific IFN-γ production and memory T cell expansion were then assessed as described above. For aim #2, the efficacy of the Rv3628 vaccine was compared with that of the BCG vaccine. Adjuvant control groups were immunized subcutaneously with dimethyldioctadecylammonium (DDA) liposomes (50 μg/injection) containing monophosphoryl lipid-A (MPL, 5 μg/injection) three times at 3-week intervals. MPL and DDA were purchased from Sigma-Aldrich (St. Louis, MO). For Rv3628 vaccination, mice were immunized subcutaneously three times at 3-week intervals with a formulation containing Rv3628 (5 μg) and MPL-DDA. The BCG-vaccinated groups were subcutaneously challenged one time with 2.0×10^5^ CFU of BCG Pasteur 1173P2 at the time of the 2^nd^ injection of the Rv3628/MPL-DDA vaccine. Three weeks after the final immunization, spleen and lung cells were harvested and used to investigate immunogenicity (i.e., IFN-γ secretion and populations of IFN-γ-producing T cells and Ag-specific IFN-γ-producing T cells). To study the protective efficacy of Rv3628, adjuvant-only (MPL-DDA) and Rv3628/MPL-DDA groups were challenged with an aerosol of the Mtb K strain as previously described [22]. Briefly, the mice were exposed to a predetermined dose of the Mtb K strain for 60 min in the inhalation chamber of an airborne infection apparatus (Glas-Col, Terre Haute, IN, USA) to expose the mice to approximately 200 CFU of viable Mtb. At 4 and 9 weeks post-challenge, spleen and lung cells were harvested from each group, and multifunctional T cells and T cell subtype populations were assessed using flow cytometry.

### Antibody titers in serum

Rv3628-specific IgG1 and IgG2c titers in serum were measured as an indicator of antigen-specific type 1 or type 2 immune responses, respectively. Briefly, plates were coated with 2 μg/ml of Rv3628, and HRP-conjugated antibodies against IgG1 or IgG2c were used as secondary antibodies. After stopping the reaction, the plates were read within 30 min at 495 nm in a microplate ELISA reader with a cut-off optical density value of 0.1.

### Bacterial counts and histopathology

The numbers of viable bacteria in the lungs and the spleens of the mice were evaluated as previously described [22]. Briefly, the bacterial count in each organ was determined by plating organ homogenates on Middlebrook 7H10 agar (Becton Dickinson, Franklin Lakes, NJ, USA) supplemented with 10% OADC enrichment medium until the late exponential phase. CFU counts were performed after 4 weeks of incubation at 37°C. Lung samples collected for histopathology were preserved overnight in 10% normal buffered formalin, embedded with paraffin, sliced into 4- to 5-mm-thick sections, and stained with hematoxylin-eosin (H&E). The superior lobes of the right lung were stained with H&E to assess the severity of inflammation. The level of inflammation in the lungs was evaluated using ImageJ software (National Institutes of Health, USA) as previously described [49].

### Statistical analysis

All *in vitro* experiments were repeated at least 3 times and produced consistent results. The data in the graphs are expressed as the means ± standard deviation. The data from the *in vivo* experiments are reported as the medians ± interquartile range (IQR). The significance of differences between two groups was determined using unpaired Student's *t*-tests, and the significance of differences between three or more groups was evaluated with one-way ANOVA followed by Dunnett's multiple comparison test using statistical software (GraphPad Prism Software, version 5.01; San Diego, CA, USA). *p < 0.05, **p < 0.01 and ***p < 0.001 were considered statistically significant.

## SUPPLEMENTARY MATERIAL FIGURES


