# Supplementary material for: Mycobacterium tuberculosis Rv3628 drives Th1-type T cell immunity via TLR2-mediated activation of dendritic cells and displays vaccine potential against the hyper-virulent Beijing K strain
Source: Oncotarget. 2016 Apr 16;7(18):24962–82. doi: 10.18632/oncotarget.8771 (PMC5041883; doi:10.18632/oncotarget.8771)
Supplement: Supplementary file 1 [file oncotarget-07-24962-s001.pdf]

Mycobacterium tuberculosis Rv3628 drives Th1-type T cell immunity *via* TLR2-mediated activation of dendritic cells and displays vaccine potential against the hyper-virulent Beijing K strain

Supplementary Material

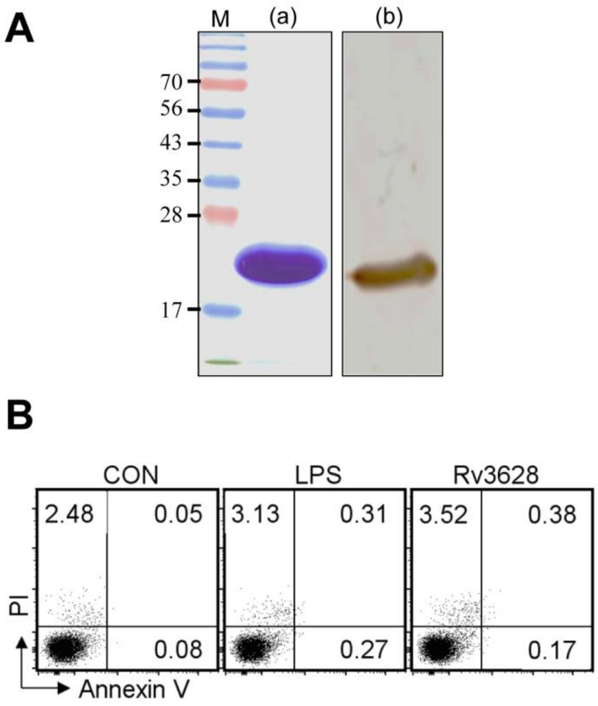

Supplementary Figure S1: Rv3628 is not cytotoxic to DCs. A. Recombinant Rv3628 protein was produced in *E. coli* BL21 cells, purified using Ni-NTA resin, and subjected to SDS-PAGE. **B.** BMDCs were analyzed by flow cytometry. Rv3628 was added on day 8, and the cultures were harvested 24 h later. The DCs were stained with anti-CD11c, Annexin V, and PI. The percentage of positive cells (Annexin V- and PI-stained cells) in each quadrant is indicated. The results of one representative experiment out of three experiments producing similar results are shown. CON: untreated DCs; LPS: LPS-treated DCs; Rv3628: Rv3628-treated DCs.

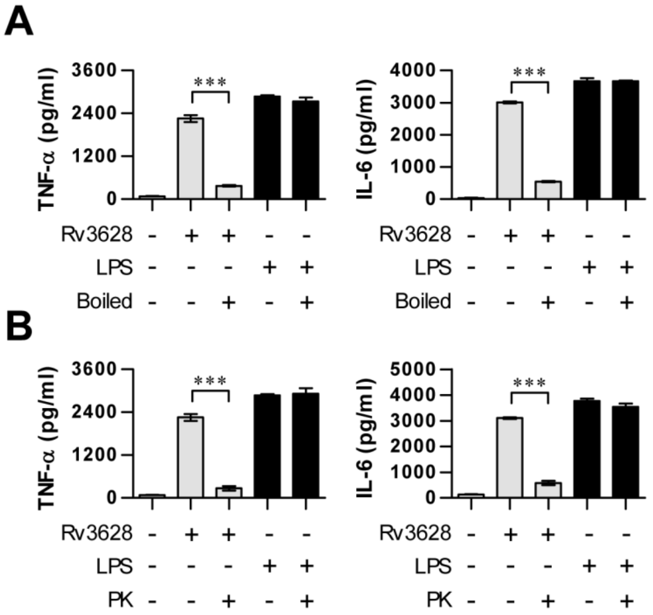

Supplementary Figure S2: Effects of endotoxins on Rv3628 protein preparation. A. and **B.** Rv3628 protein or LPS was heated for 30 min at 100°C or digested with proteinase K (PK, 500 ng/ml) and added to DCs as described above. The amounts of TNF-α and IL-6 in the culture medium were measured by ELISA. One representative plot out of three independent experiments is shown. All data are expressed as the means ± SD of 3 samples. ****p* < 0.001 indicates statistical significance compared with DCs treated with Rv3628 alone.

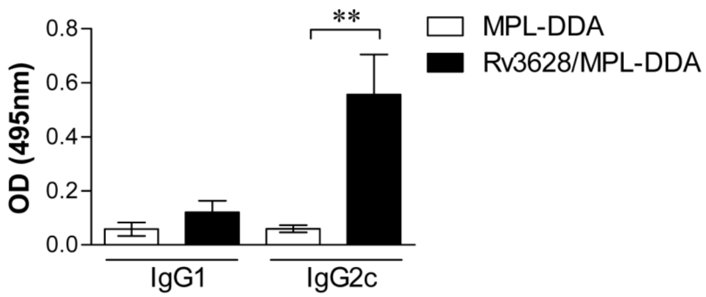

Supplementary Figure S3: Antibody response in serum after the final immunization with MPL-DDA alone or Rv3628/MPL-DDA. Serum samples were analyzed by ELISA for Rv3628-specific IgG1 and IgG2c antibodies in each group of mice. Data from one of two experiments producing similar results are shown (n = 6 animals/group). **$p < 0.01$ compared with the MPL-DDA-alone group (unpaired t-test).

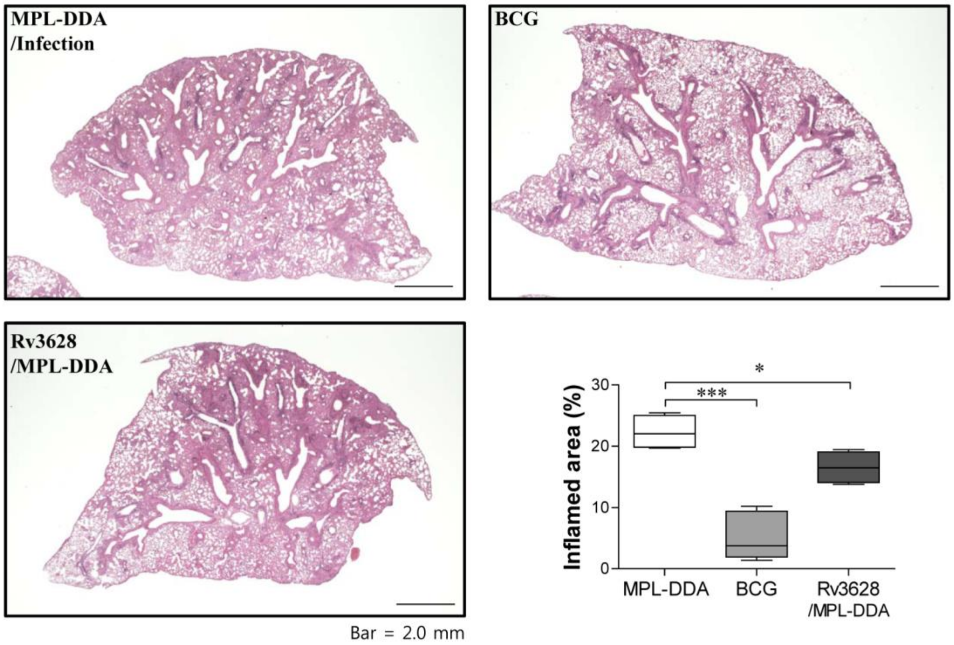

Supplementary Figure S4: Histopathological lesions of the lung at 4 weeks post-infection. Lung sections from each immunized mouse (immunization with MPL-DDA alone, BCG alone or Rv3628/MPL-DDA) were stained with H&E at 4 weeks post-challenge with Mtb K. Data from one of two experiments producing similar results are shown (*n* = 6 animals/group). *$p < 0.05$ and ***$p < 0.001$ compared with the MPL-DDA-alone group.

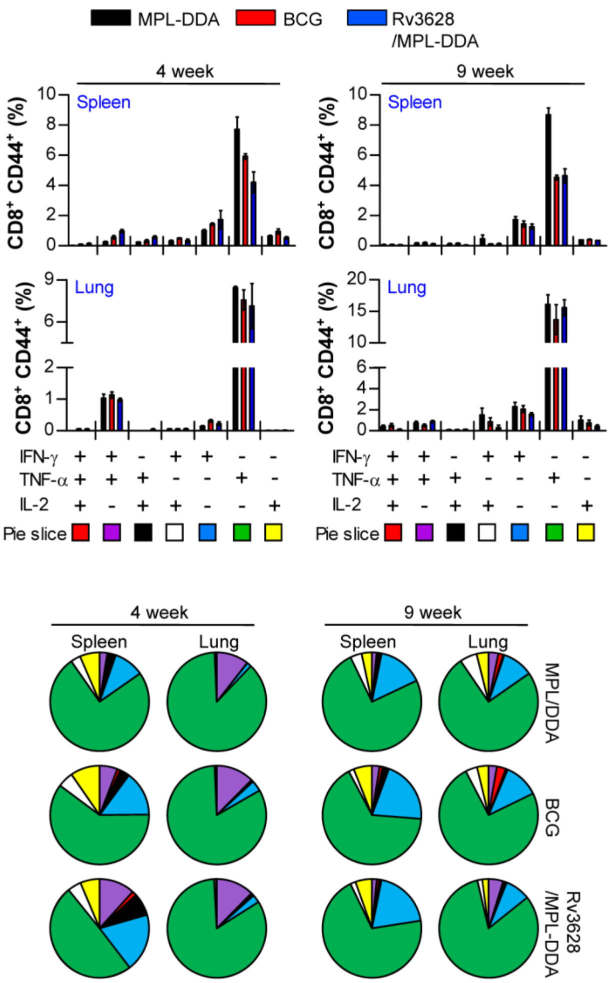

Supplementary Figure S5: Rv3628-specific multifunctional CD8$^+$ T cells were analyzed by flow cytometry at 4 and 9 weeks post-infection. Spleen and lung cells were isolated from mice immunized with Rv3628/MPL-DDA at 4 and 9 weeks post-infection and were stimulated with Rv3628 before flow cytometry. The percentages of cells expressing all three cytokines (IFN-γ, TNF-α, and IL-2), two of these three cytokines, or one of these three cytokines in each group are depicted in the bar graphs (top panel) and pie charts (bottom panel). The gating strategy was identical to that described in Figure 10A. The results of one representative study out of at least two independent studies are presented.

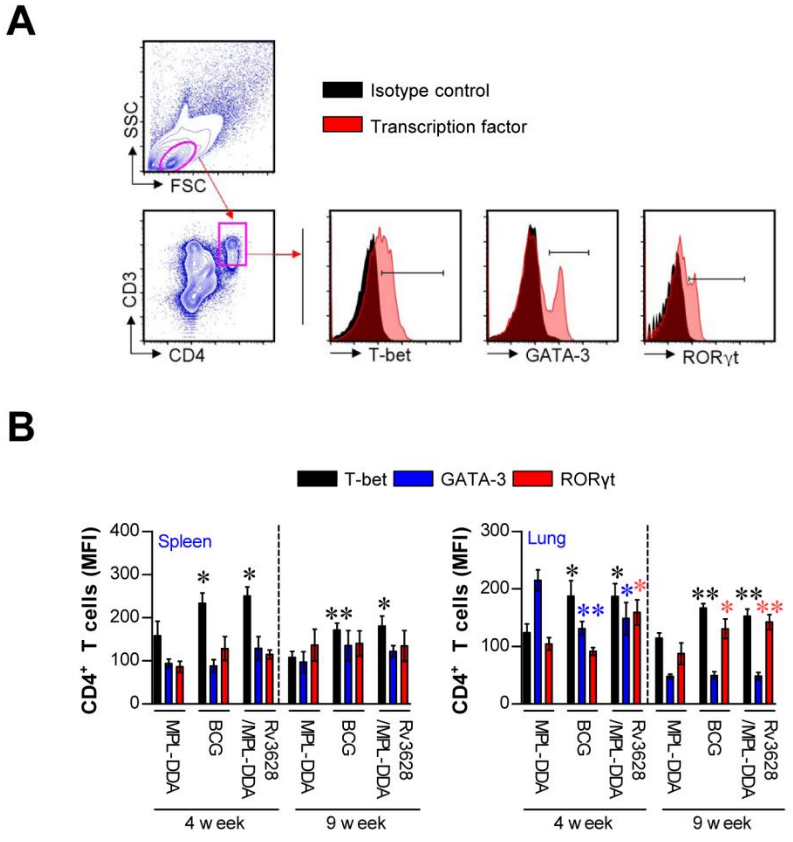

Supplementary Figure S6: Rv3628/MPL-DDA-immunized mice produce Th1 and Th17 cells after challenge with Mtb. A. Gating strategy used to identify the principal $CD4^+$ T cell subset. **B.** The bar graphs show the expression of transcription factors such as T-bet, GATA-3 and RORγt in $CD3^+CD4^+$ cells among spleen and lung cells isolated from Mtb K-infected mice. Data from one of two experiments producing similar results are shown (n = 6 animals/group). *$p < 0.05$, **$p < 0.01$, and ***$p < 0.001$ compared with the MPL-DDA-alone group (unpaired t-test).
